# Digging into the Atomistic Details of the TaN/MgO Interface: An Ab Initio Study Supported by Transmission Electron Microscopy

**DOI:** 10.1021/acsmaterialsau.4c00173

**Published:** 2025-01-28

**Authors:** Victor Quintanar-Zamora, Joseph P. Corbett, Rodrigo Ponce-Pérez, Armando Reyes Serrato, Carlos Antonio Corona-Garcia, Oscar Contreras-López, Jonathan Guerrero-Sanchez, Jesús Antonio Díaz

**Affiliations:** † Posgrado en Nanociencias, Centro de Investigación Científica y de Educación Superior de Ensenada, Baja California, Ensenada, Baja California 22860, México; ‡ Centro de Nanociencias y Nanotecnología, Universidad Nacional Autónoma de México, Ensenada, Baja California 22860, México; § Department of Physics, College of Arts and Science, Miami University, 501 E High St, Oxford, Ohio 45056, United States

**Keywords:** tantalum nitride, magnesium oxide, interface, TEM, DFT, thermodynamic stability

## Abstract

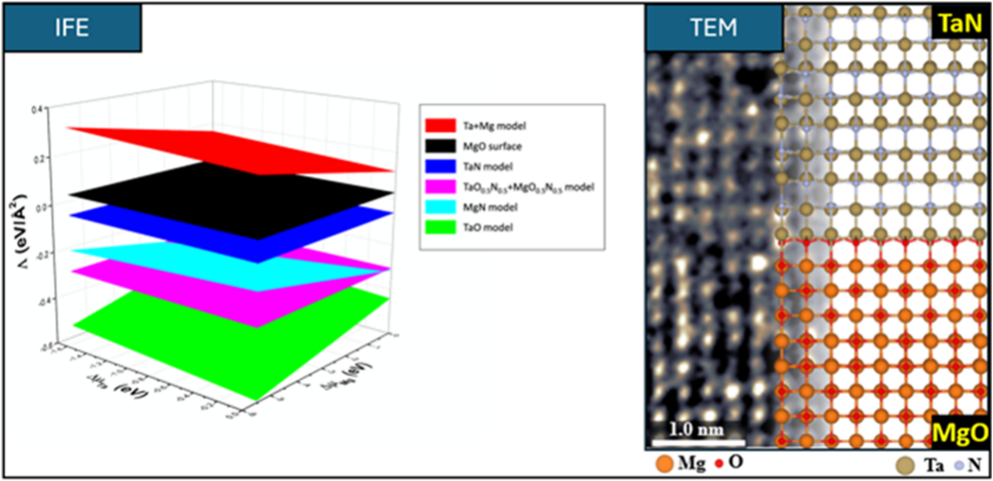

First-principles calculations of the TaN/MgO (001) interface were performed within the DFT framework. A thermodynamic stability analysis identified four stable interfaces. The most stable configuration for the interface consists of a TaO monolayer formed between the TaN and MgO layers. The density of states at *E*_F_ indicates that all interface models exhibit metallic behavior. The electron localization function reveals that all of these models exhibit ionic-type bonds at the interface. In addition to the computational simulations, epitaxial growth of the TaN thin films on FCC MgO (001) substrates was carried out by using pulsed laser deposition. Transmission electron microscopy images of the TaN/MgO (001) interface cross-section reveal that TaN film grows on the MgO substrate following the epitaxial relationship TaN [001] || MgO [001]. An FFT analysis of the TaN films demonstrates that the TaN lattice contracts at the interface with MgO conforming to the substrate lattice, corroborating the computational predictions. Our results provide evidence that strained TaO layers mediate the TaN/MgO (001) interface formation.

## 1 Introduction

Tantalum nitride is a material classified as refractory nitride because of its high hardness and high melting point.^1^ It also exhibits metallic characteristics such as high thermal and electrical conductivity.^2^ Besides, the electrical properties of tantalum nitride thin films can range from metallic to insulating behavior, depending on the amount of nitrogen in the compound.^3^

Tantalum nitride exhibits a wide variety of crystal phases; the majority are nonsuperconductive, which include the γ-Ta_2_N and ε-TaN,^4^ Ta_5_N_6_, Ta_4_N_5_,^5^ and Ta_3_N_5_.^6^ θ-TaN phase presents superconductivity at a critical temperature (*T*_c_) of 3.1 K but only when a pressure of 24.6 GPa is applied.^7^ The δ-TaN_1–*x*_ is the only phase of tantalum nitride that exhibits superconductivity at ambient pressure,^8^ at a maximum *T*_c_ of 10.8 K;^9^ this phase crystallizes in the NaCl-type structure corresponding to the *Fm*3̅*m* space group.^10^

Some applications of δ-TaN_1–*x*_ (for now on TaN) are in superconducting films and devices such as superconducting nanowire single-photon detectors (SNSPD),^11^ superconductor–normal metal–superconductor (SNS) Josephson junctions,^12^ normal metal–insulator–superconductor (NIS) tunnel junctions,^13^ superconducting spintronics for its quasiparticle-mediated inverse spin Hall effect (QMiSHE),^14^ spin–orbit torques (SOTs),^15^ diffusion barrier in ultra large scale integration (ULSI) circuits,^16^ among others.

Among the explored substrates for superconducting TaN thin film deposition, those grown on MgO have exhibited higher-quality growth;^17^ this can be attributed to the small lattice mismatch of 3.09% between TaN (*a* = 4.34 Å^10^) and MgO (*a* = 4.21 Å^18^). Film–substrate systems with a low lattice mismatch are more feasible to form heteroepitaxial interfaces. Thus, TaN thin films can be growing epitaxially under compressive stress on (001) MgO surfaces; although the lattice mismatch is relatively small, the TaN structure near the interface might be suffering lattice contraction. However, the atomic arrangement at the TaN/MgO interface has not been extensively studied. Therefore, we performed *ab initio* calculations to explore it in detail.

Understanding the formation of the TaN/MgO interface under real growth conditions is essential to develop further applications in superconducting devices based on TaN thin films. This work presents the simulation of several interface models using density functional theory (DFT) calculations to determine the structural, thermodynamic, and electronic properties of the TaN/MgO (001) interface at the atomic scale. Additionally, it includes the experimental growth of TaN thin films on MgO (001) substrates, transmission electron microscopy (TEM) characterization, and k-spacing analysis using a fast Fourier transform (FFT) of local atomic resolution to demonstrate the epitaxial growth between the film and the substrate. We demonstrate theoretically and experimentally that TaN is compressed at the interface by the substrate effect and that TaO layers could mediate interface formation.

## 2 Methods

**Figure 1:**
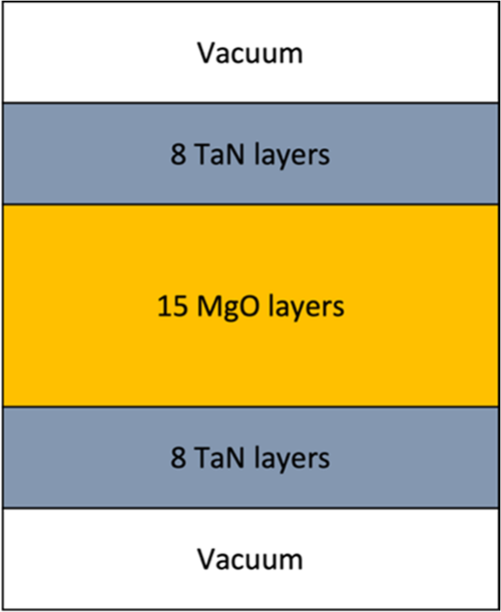
Schematic diagram of the supercell used to simulate the different TaN/MgO interface models.

The structural, thermodynamic, and electronic properties of the TaN/MgO (001) interface have been investigated at the atomic scale by first-principles calculations. Simulations were performed within the DFT framework as implemented in the Vienna Ab initio Simulation Package (VASP)^19−22^ code. The electron–ion interactions were treated using the projector augmented wave basis (PAW)^23^ with an energy cutoff of 520 eV. The exchange-correlation energy is treated according to the generalized gradient approximation (GGA) with the Perdew–Burke–Ernzerhof (PBE) parametrization.^24^ The TaN/MgO (001) interface was simulated using the supercell method considering a slab with 1 × 1 periodicity. Along the supercell *c*-axis, which is aligned with TaN and MgO < 001> direction, a slab of 15 MgO monolayers (ML) thick is stacked between 2 TaN slabs of 8 ML thick each one. The supercell was completed by adding a vacuum region below and above the TaN/MgO/TaN structure to avoid interactions between the adjacent slabs. This specific crystal arrangement defines a center-symmetric supercell simulating a MgO bulk-like substrate interfacing with two TaN ultrathin films with their respective surfaces (see Figure 1). The Brillouin zone was sampled using the special point scheme of Monkhorst–Pack^25^ with a *k*-points grid of 7 × 7 × 1. In geometry optimization, convergence is reached when the energy differences are less than 1.0 × 10^–4^ eV and all the force components are less than 0.01 eV/Å.

The computational simulation was accompanied by the experimental growth of 35 nm TaN thin films on FCC MgO (001) using pulsed laser deposition (PLD); the procedure was described in our previous work.^26^ A TEM analysis in cross-sectional view of the TaN/MgO interface was performed with a JEOL JEM-2100F scanning transmission electron microscope (STEM), provided with a 200 keV Schottky-type field emission electron gun. A lamella from the TaN/MgO sample was prepared with a dual beam system scanning electron microscope-focused ion beam (SEM-FIB) JEOL JIB-4500; the TEM sample was thinned below 100 nm in thickness by micromilling with gallium ions. The specimen was analyzed in a conventional parallel-beam mode. Cross-section high-resolution micrographs of the TaN thin film and MgO substrate were acquired along the [100] zone axis. The interplanar spacing along the in-plane (IP) and out-of-plane (OOP) crystal directions with respect to the substrate normal was estimated through k-spacing analysis from FFT of local atomic resolution.

## 3 Results and Discussion

### 3.1 Atomic-Scale Analysis

**Figure 2:**
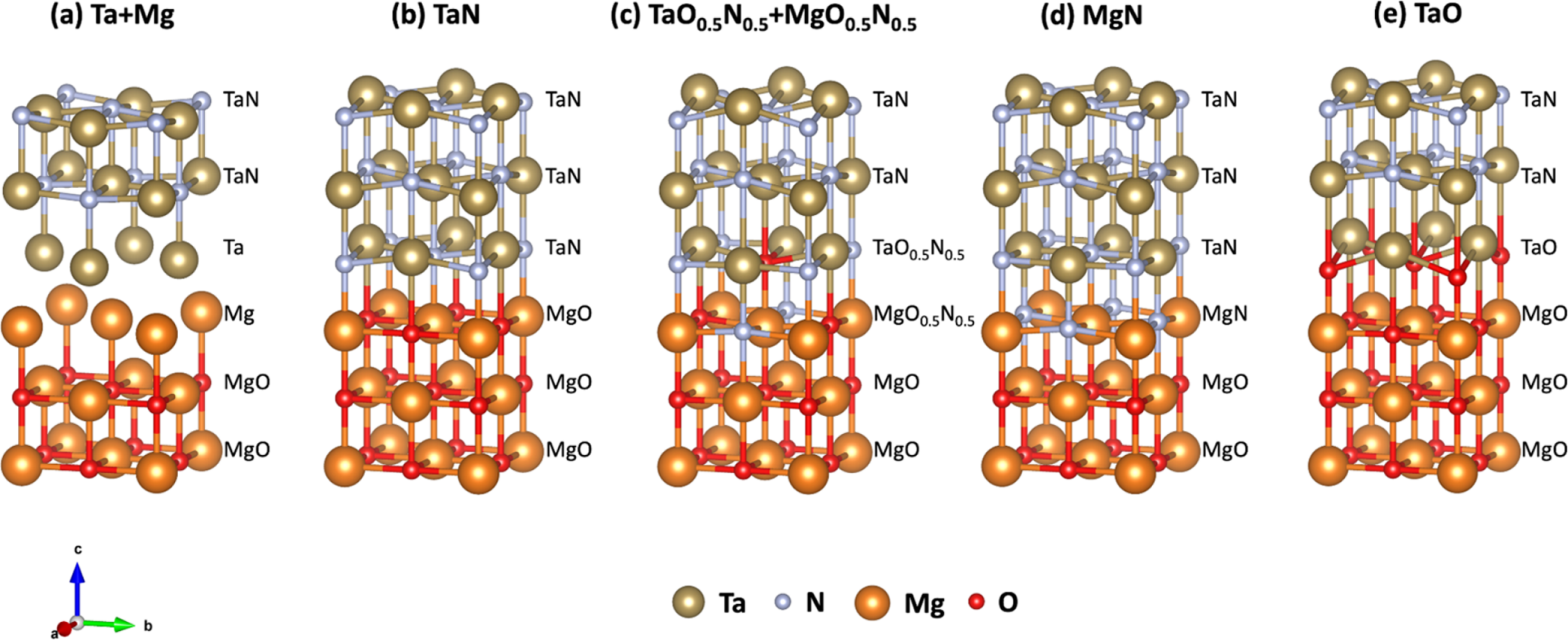
Structural models for the TaN/MgO (001) interface (a) Ta+Mg, (b) TaN, (c) TaO_0.5_N_0.5_+MgO_0.5_N_0.5_, (d) MgN, and (e) TaO.

The TaN/MgO (001) interface is studied on the atomic scale by considering different interface models that preserve the rock-salt structure, as observed in the experiment. Figure 2 shows the five most stable models. The considered models in Figure 2 are named according to their distinctive layers located at the interface: Ta+Mg, TaN, TaO_0.5_N_0.5_+MgO_0.5_N_0.5_, MgN, and TaO. Each interface model has one or two layers with atomic arrangements different from those of the film and substrate, except for the TaN model, which represents the ideal arrangement of a TaN film on a MgO substrate without any modifications at the interface. Regarding the colors of the atoms in the structural models, dark yellow is Ta, blue is N, orange is Mg, and red is O.

Both materials crystallize in the rock-salt crystal structure after relaxation, with calculated lattice parameters of 4.25 and 4.42 Å for MgO and TaN, respectively, in good agreement with previous reports.^27,28^ The calculated Mg–O bond distance is 2.13 Å, while the Ta–N distance is 2.21 Å. The lattice mismatch between the unit cells of TaN and MgO is 4.00%, a value closer to the experimental lattice mismatch of 3.09%. This low mismatch ensures epitaxial growth and allows us to simulate the TaN/MgO interface using first principles.

Before simulating the interfaces, we fully relaxed the ideal-terminated MgO (001) surface, which exhibits two types of Mg–O bonds at the surface: parallel and perpendicular. The parallel Mg–O bonds contract to 2.11 Å, compared to the 2.13 Å in the MgO bulk, whereas the perpendicular Mg–O bonds either expand or contract depending on the surface-exposed atom. When O is the exposed atom, the bond length increases to 2.15 Å; in contrast, when Mg is the exposed atom, the bond length contracts to 2.11 Å; this behavior agrees with the results reported by Alam et al.^29^ Also, the interplanar spacing of layers close to the MgO surface is contracted compared with the MgO bulk region of the slab.

Once the different TaN/MgO (001) interface models are relaxed (see Figure 2) we notice that the parallel Ta–N bonds of all models in the second film layer at the (100) plane exhibit a contraction compared to the calculated TaN bulk, decreasing from 2.21 Å to 2.17, 2.18, 2.19, 2.20, and 2.15 Å in the Ta+Mg, TaN, TaO_0.5_N_0.5_+MgO_0.5_N_0.5_, MgN, and TaO models, respectively.

Furthermore, the Ta+Mg model’s interlayer distance is 2.10 Å between TaN and Ta layers, 1.96 Å between Ta and Mg layers, and 2.19 Å between Mg and MgO layers. For the TaN model, the interlayer distance is 2.23 Å between the TaN and MgO layers. For the TaO_0.5_N_0.5_+MgO_0.5_N_0.5_ model, the interlayer distance is 2.54 Å between the upper TaN layer and the TaO_0.5_N_0.5_ layer, 2.15 Å between the TaO_0.5_N_0.5_ and MgO_0.5_N_0.5_ layers, and 2.16 Å between the MgO_0.5_N_0.5_ layer and the lower MgO layer. For the MgN model, the MgN layer shows an interlayer distance with the upper TaN layer and the lower MgO layer of 2.12 and 2.17 Å, respectively. For the TaO model, the TaO layer exhibits an interlayer distance with the upper TaN layer and the lower MgO layer of 2.67 and 2.33 Å, respectively.

### 3.2 Thermodynamic Stability

The thermodynamic stability of the TaN/MgO (001) interface is analyzed by simulating several interface models with different types and amounts of atoms. Therefore, the Interface Formation Energy (IFE) formalism^29,30^ must be applied. In our case, the energy of the TaN/MgO slab is defined as follows
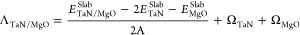
1Where *E*_TaN/MgO_^Slab^, *E*_TaN_^Slab^, and *E*_MgO_^Slab^ are the total energies in eV of the TaN/MgO system, isolated TaN slab, and isolated MgO slab, respectively; A is the area of the interface (in Å^2^); Ω_TaN_ and Ω_MgO_ are the surface formation energies of each compound, defined as
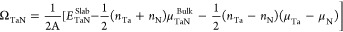
2and
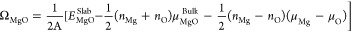
3where *n_i_* and μ*_i_* are the number of atoms and the chemical potential of the *i*th species, respectively.

**Figure 3:**
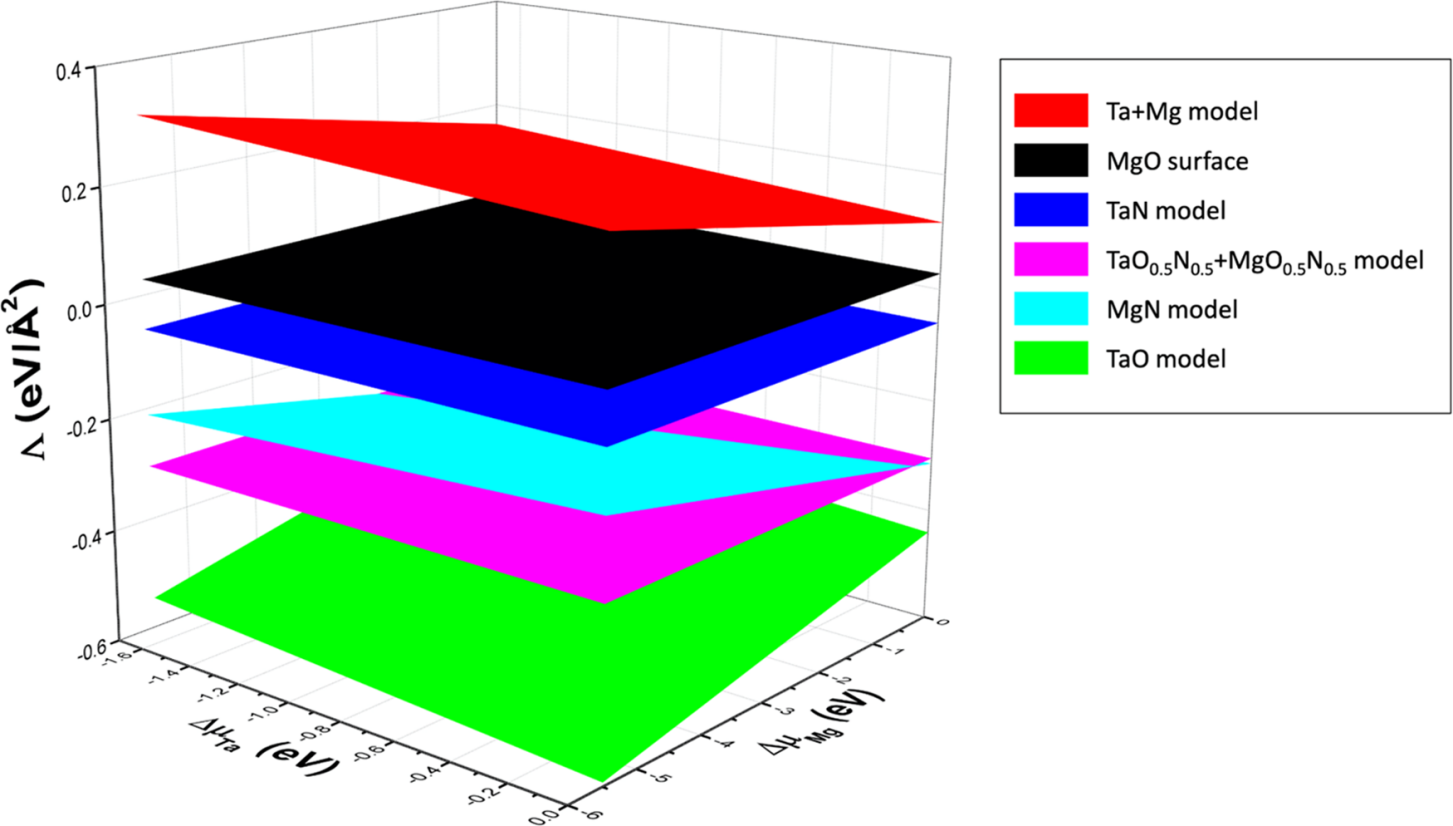
IFE vs Δ*μ*_Ta_ vs Δ*μ*_Mg_ of the Ta+Mg, TaN, TaO_0.5_N_0.5_+MgO_0.5_N_0.5_, MgN, and TaO models.

A 3D plot in Figure 3 shows the interface formation energy versus Ta and Mg chemical potentials. Each plane represents a different interface model. For TaN, the chemical potential ranges from Ta-rich conditions (μ_Ta_ = μ_Ta_^Bulk^) to Ta-poor conditions (μ_Ta_ = μ_Ta_^Bulk^–μ_N_^Mol^). For MgO, the chemical potential varies from Mg-rich conditions (μ_Mg_ = μ_Mg_^Bulk^) to Mg-poor conditions (μ_Mg_ = μ_Mg_^Bulk^–μ_O_^Mol^). According to the IFE formalism, the most stable interface has the lowest Λ value.

As a reference, we include the surface formation energy of the ideal-terminated MgO (001) surface (black plane), which provides a value of 0.03 eV/Å^2^ for the entire range of chemical potentials. Notice that the Ta+Mg interface model (red plane) has larger values than the bare MgO surface, denoting its poor stability.

Although the TaN interface model (blue plane), TaO_0.5_N_0.5_+MgO_0.5_N_0.5_ model (magenta plane), and MgN model (cyan plane) provide lower IFE values than the bare MgO (001) surface results to be unstable in comparison with the TaO interface (green plane) model which provides the lowest Λ values for the entire range of chemical potential from Ta-rich to Ta-poor and Mg-rich to Mg-poor conditions, establishing it as the most stable model among all those considered. This TaO model represents the oxidation of TaN when it is deposited on MgO, which is consistent with our previous predictions that oxygen incorporation into TaN is thermodynamically favorable.^26^ Hence, a monolayer of TaO appears when the O atoms replace the N atoms at the interface, concluding that the epitaxial growth of TaN onto MgO (001) substrates is mediated by the formation of TaO monolayers at the interface.

The oxygen in the TaO layer could be acquired during growth by substituting N atoms in the first layer of the film with O atoms or by substituting Mg atoms in the first layer of the substrate with Ta atoms. Experimentally, the TaN epitaxial growth is performed under Ta-rich conditions, so it is more probable that Ta replaces Mg at the interface. According to the IFE calculations, under Ta-rich conditions, the TaO interface can be formed over the entire range of the Mg chemical potential, indicating that the growth of TaO at the interface is feasible.

### 3.3 Electronic Properties

**Figure 4:**
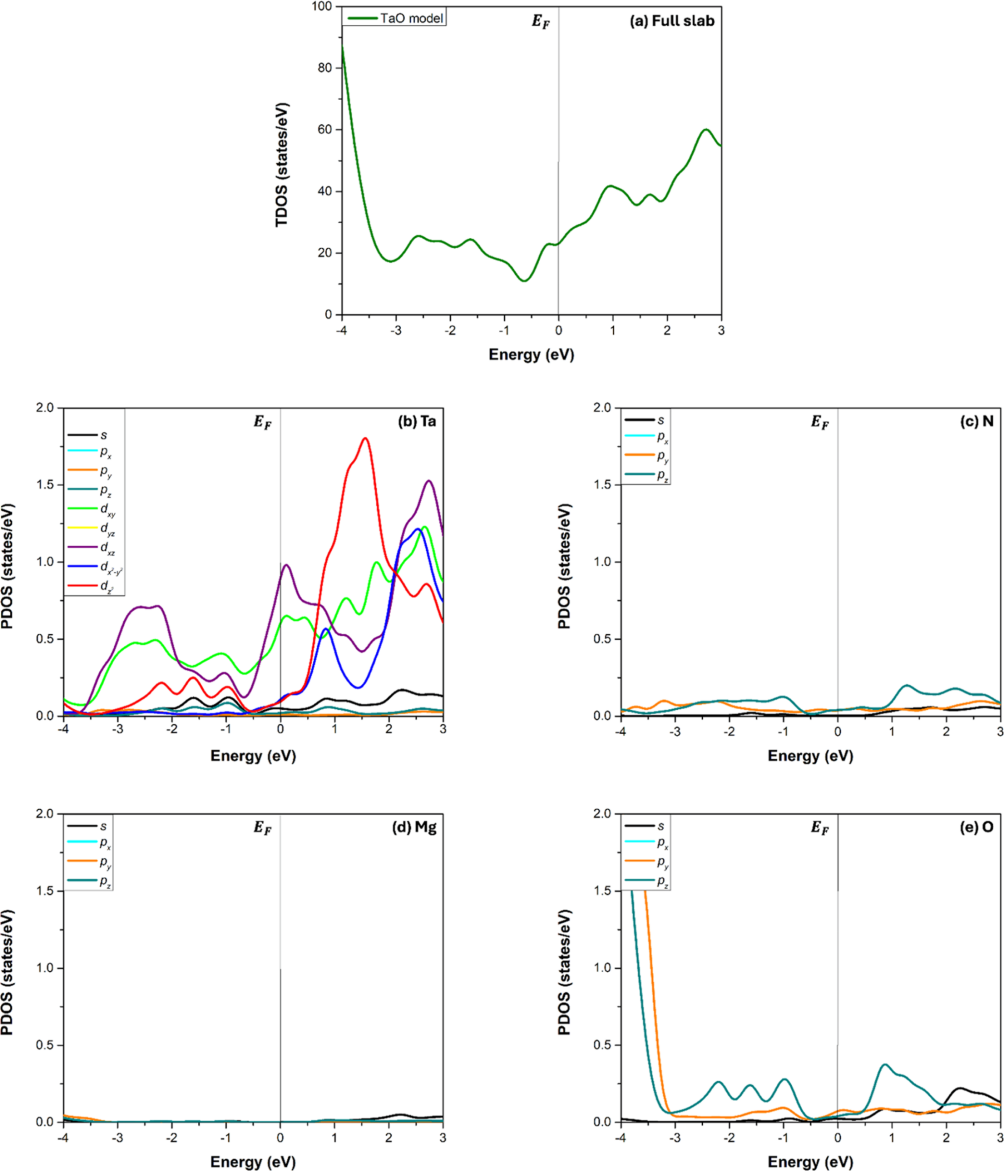
Density of states of the TaO model: (a) total DOS of the full slab and projected DOS of the (b) Ta, (c) N, (d) Mg, and (e) O atoms at the interface layers.

We investigated the electronic properties of the TaN/MgO interface models by calculating their density of states (DOS) and electron localization function (ELF). Figure 4 shows the total DOS of the stable TaO interface model along with the projected DOS of the two TaN layers and the two MgO layers closest to the interface. In all graphs, the energy reference is the Fermi level (*E*_F_). The total DOS shows a continuous density of states around the *E*_F_ in Figure 4a; thus, the system is metallic. The projected DOS at the interface indicates that the major contribution at the *E*_F_ is due to Ta atoms in Figure 4b, particularly to the degenerated d_*xz*_ and d_*yz*_ orbitals. The next significant contribution is due to the d_*xy*_ orbital, followed by the d_*x*^2^–*y*^2^_ and d_*z*^2^_ orbitals. The subsequent minor contributions are due to the s orbital, the p_*z*_ orbital, and the degenerated p_*x*_ and p_*y*_ orbitals. The N atom’s contribution to the DOS at the *E*_F_ is lower, as seen in Figure 4c, where the main contribution is due to the p_*z*_ orbital, followed by the degenerated p_*x*_ and p_*y*_ orbitals, and finally, the s orbital. In addition, Mg contribution to the *E*_F_ is nearly zero, see Figure 4d; this behavior is attributed to the insulating nature of the MgO substrate. In the case of O atoms, see Figure 4e, their contribution to the DOS around the *E*_F_ is due to the degenerated p_*x*_ and p_*y*_ orbitals, then the p_*z*_ orbital, and finally, the s orbital.

The total DOS and projected DOS of the Ta+Mg, TaN, TaO_0.5_N_0.5_+MgO_0.5_N_0.5_, and MgN models are shown in Supporting Information. In all cases, the interface models exhibit a continuous density of states at *E*_F_, similar to that of the TaO model. Based on these findings, we conclude that the d-orbitals of the Ta atoms control the metallic behavior of all the interface models.

**Figure 5:**
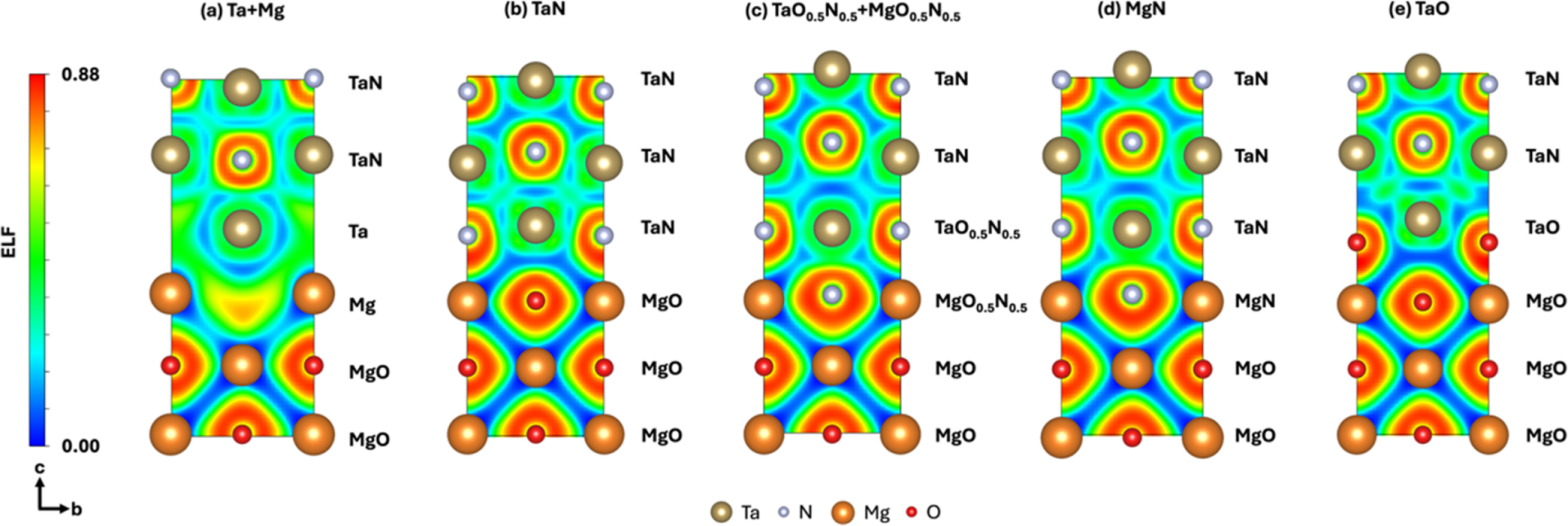
ELF maps at the (100) plane of the (a) Ta+Mg, (b) TaN, (c) TaO_0.5_N_0.5_+MgO_0.5_N_0.5_, (d) MgN, and (e) TaO models.

Figure 5 shows the ELF maps at the (100) plane of the five TaN/MgO interface models. For simplicity, this figure shows only three layers of the film and three layers of the substrate. A color map is plotted from 0 to 0.88, representing the probability of finding an electron. Red corresponds to electron accumulation, blue corresponds to electron depletion, and yellow, green, and cyan correspond to intermediate values. Notice that TaN and MgO exhibit ionic-type bonds, but the ionic character in MgO is stronger than that in TaN.

The electrons are mainly localized at the most electronegative atoms, such as those of O and N. In contrast, the electrons exhibit an intermediate probability of being localized at Ta atoms and a low probability of being localized at Mg atoms. The difference between the several interface models is only in the shape of the electron distribution and the atomic positions, but the ionic behavior remains. The Ta+Mg model confers intermediate electron accumulation to the N-vacancies located at the Ta layer and to the O-vacancies located at the Mg layer. Still, this model is unstable, as demonstrated by our interface formation analysis.

**Figure 6:**
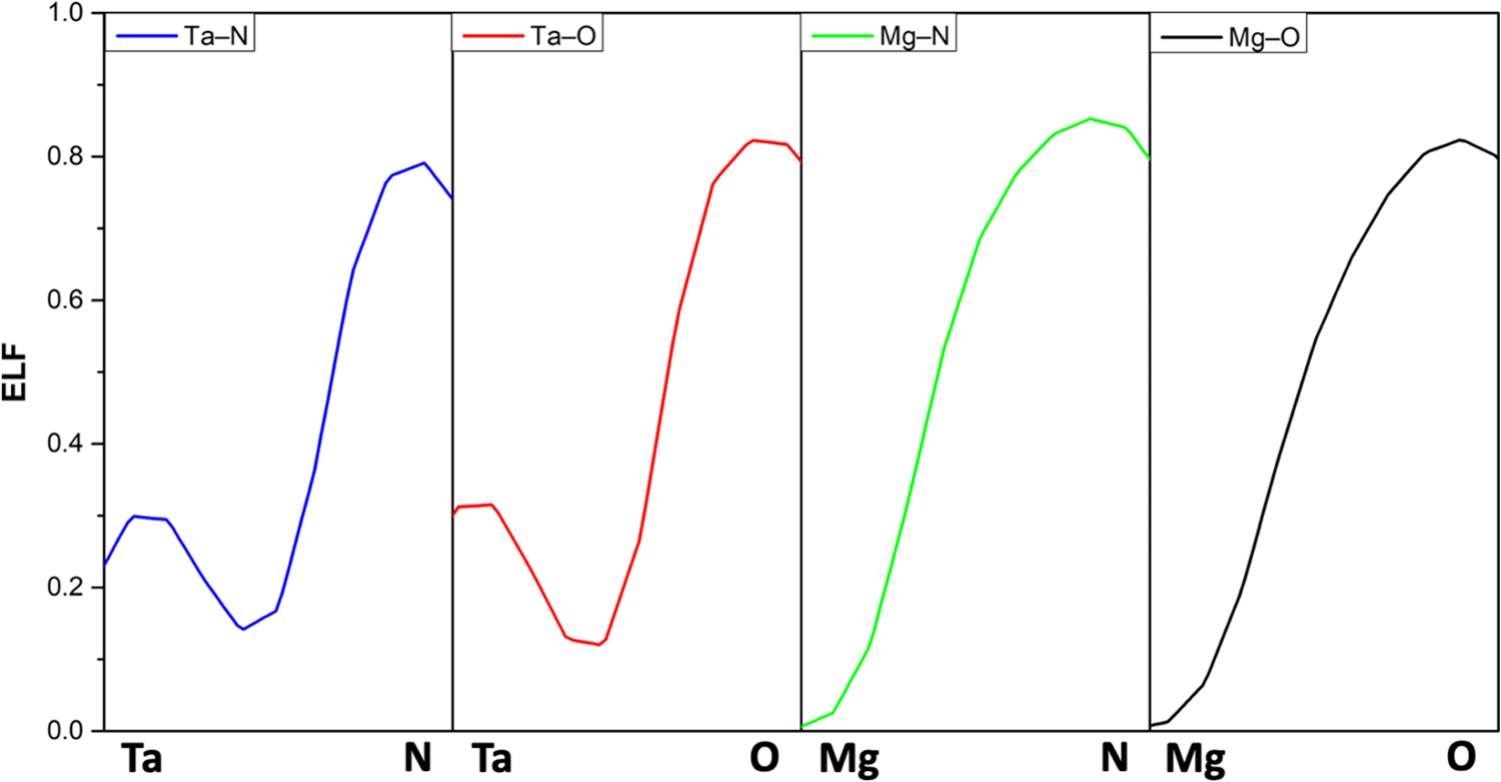
ELF line profiles of the Ta–N, Ta–O, Mg–N, and Mg–O bonds.

Finally, Figure 6 shows the ELF line profiles for the Ta–N, Ta–O, Mg–N, and Mg–O bonds at the interface in the TaN model, as all four possible bonds are present in this system. It is confirmed that O and N exhibit high ELF values, Ta shows intermediate ELF values, and Mg displays the lowest values. The line profile shapes indicate that all bonds are mainly ionic.

### 3.4 Crystal Structure and Interface

**Figure 7:**
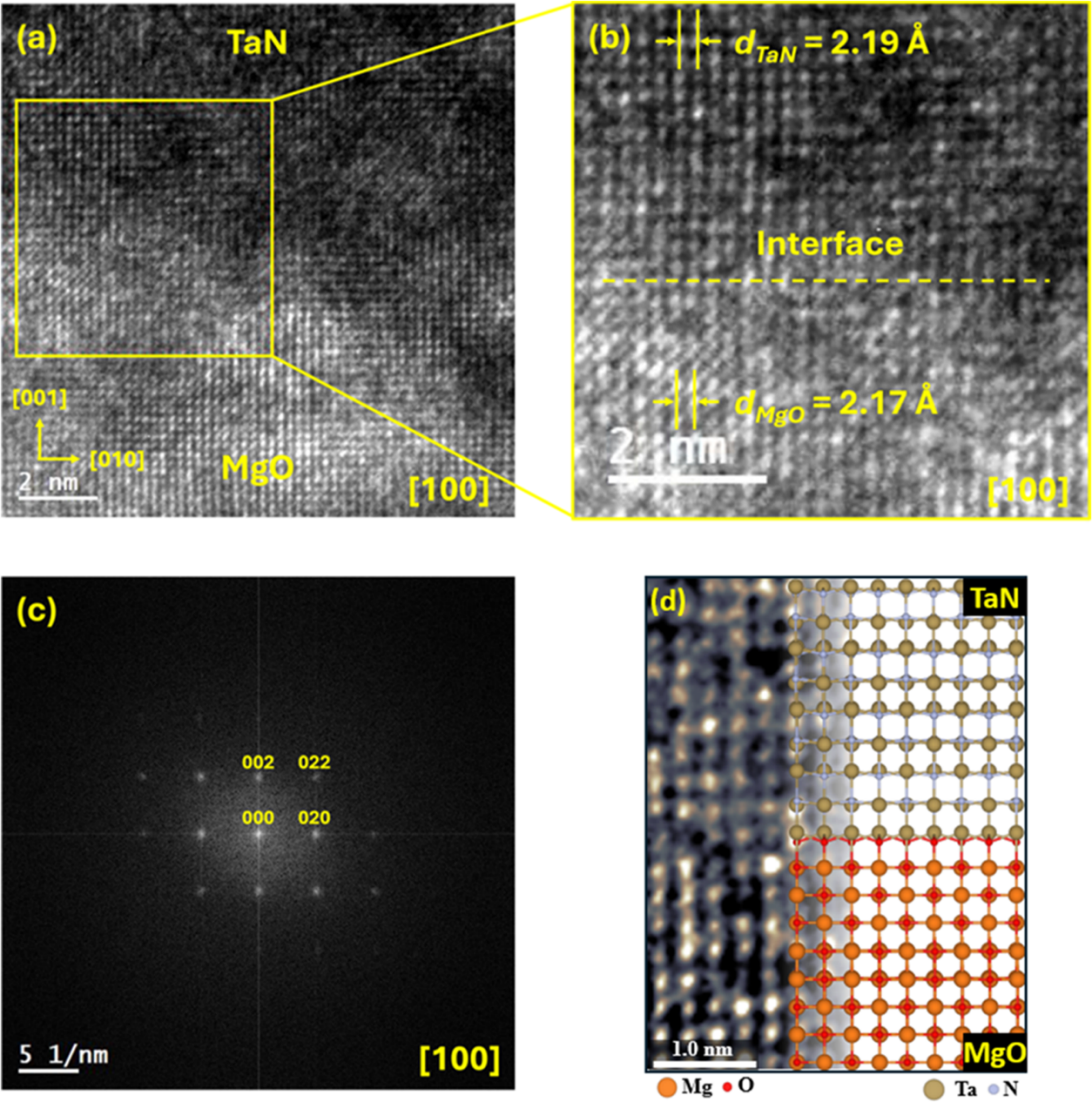
TEM analysis of the TaN thin film and MgO substrate. (a) Cross-section micrograph of the TaN/MgO interface, (b) 2x enlarged section of the micrograph to visualize the interface, (c) FFT image of the TaN/MgO interface, and (d) Atomic-scale overlay of the lowest energy model on the atomically resolved micrograph.

The TEM imaging was performed in bright field mode on a lamella cross-section of the TaN/MgO (001) sample; observations were carried out along the [100] zone axis, as shown in Figure 7a. In this HRTEM micrograph, the TaN/MgO interface is identified by the image contrast established between both materials; the TaN region (top) is observed with less intensity (darker) due to a Z contrast effect. A 2x enlarged micrograph of the TaN/MgO interface is presented in Figure 7b, and its corresponding FFT image is shown in Figure 7c. A local FCC structure for both compounds is estimated from the positions of the Mg and Ta atoms in the HRTEM micrograph and is corroborated in the FFT image, where the *k*-points correlate with the lattice fringes observed in the HRTEM micrograph and are indexed to the (002), (020), and (022) planes of the FCC structure. The enlarged section of the micrograph, where the interface is observed, demonstrates an atomically sharp interface and excellent epitaxial growth on the MgO substrate; from this image, the epitaxial relationship TaN [001] || MgO [001] is established. Furthermore, the lowest energy interface model (previously discussed) of the TaN/MgO heterostructure overlaid atop the atomically resolved micrograph in Figure 7d demonstrates excellent agreement between our experimental observations and the theoretical lowest energy model.

**Figure 8:**
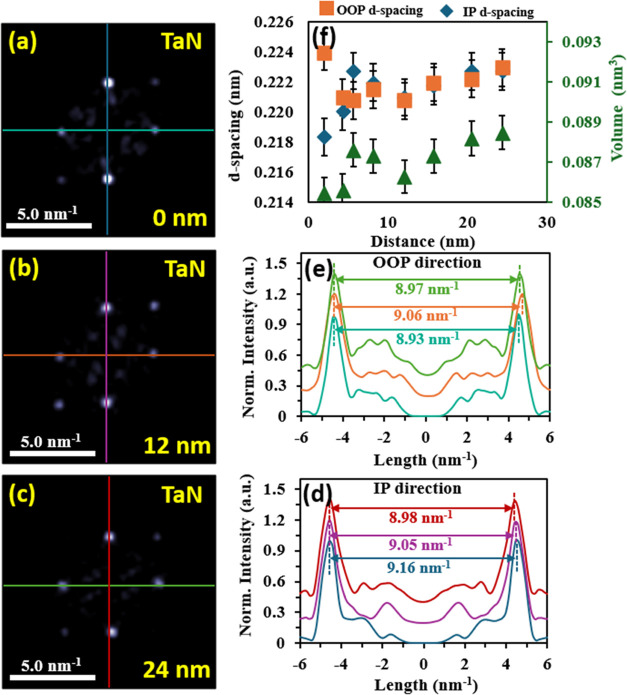
FFT analysis and line cuts as a function of distance from the TaN/MgO interface. (a–c) selective area FFT images from 6.25 nm^2^ windows at distances 0, 12, and 24 nm from the interface, respectively. Corresponding line cuts of the *k*-points are shown in (d, e) and color-coded for the OOP and IP directions. (f) computed *d*-spacing and lattice volume from k-spacing measurements.

To assess the interfacing and relaxation of the TaN on the MgO substrate, selective area FFT analysis is performed using a 6.25 nm^2^ analysis window where several FFT images are produced as a function of distance from the interface, see Figure 8. The IP and the OOP interplanar spacings are extracted from the separation of mutual *k*-points corresponding to the respective directions. The interplanar spacing for both directions as a function of distance is shown in Figure 8f, where at the interface, a contraction of the TaN IP *d*-spacing to 2.18 ± 0.015 Å, is observed, while the OOP spacing is 2.22 ± 0.015 Å. The IP *d*-spacing undergoes an appreciable relaxation up to 2.23 ± 0.015 Å, whereas the OOP *d*-spacing undergoes a slight relaxation to 2.23 ± 0.015 Å. In particular, after ∼10 nm, both IP and the *d*-spacing of the OOP converge to a square lattice and mutually relax to 2.23 Å. As an additional measure of the strain, we compute the change in volume of the lattice using the IP and the OOP *d*-spacing and plot this alongside the *d*-spacing color-coded in green for the secondary *y*-axis. We observe an increase of the lattice volume as the film thickness, with a maximal volume change of 0.0031 ± 0.00082 nm^3^ or approximately 3% change from the interface to 24 nm up the film. Figure 8a–c shows the selection of FFT images with corresponding line cuts of Figure 8d,e along this relaxation trend at the interface, ∼10 nm from the MgO interface and ∼25 nm surface interface. The line cuts of Figure 8d,e are color-coded to FFT images in Figure 8a–c. The line cuts are aligned to the left *k*-point, and a peak-to-peak distance is measured from fitting Gaussian functions to the particular *k*-point. The extracted peak-to-peak distances are color-coded and labeled on the line cuts. This series visually demonstrates the relaxation in the *k*-points from a slightly distorted cubic structure to a relaxed cubic structure.

The results obtained through TEM images and FFT analysis demonstrate that the TaN lattice contracts at the interface with MgO conforming to the substrate lattice with a difference of only ∼0.01 Å. In contrast, the interplanar spacing difference between the individual bulks of TaN^10^ and MgO^18^ is 0.065 Å.

## 4 Conclusions

First-principles calculations based on density functional theory reveal that epitaxial growth in the TaN/MgO (001) interface is feasible by the formation of TaO at the interface. The interface exhibits metallic behavior, as confirmed by the DOS calculations. The projected DOS indicates that the most significant contribution at the Fermi level comes from Ta-*d* orbitals. All models exhibit ionic bonds at the interface, as evidenced by the electron localization function maps; the TaN film and the MgO substrate are also ionic with a stronger ionic character in MgO. In addition, TaN thin films were grown epitaxially on MgO substrates. A local FCC structure was estimated for both materials by transmission electron microscopy analysis. Substrate and film exhibit a close interplanar spacing at the interface and present a local epitaxial growth in the [001] direction of the substrate, which corresponds to the epitaxial relation TaN [001] || MgO [001], as demonstrated in the TEM micrograph and FFT analysis. Our FFT analysis also indicates that the TaN lattice contracts at the interface with the MgO substrate, corroborating the computational predictions.

## Data Availability

Data will be made available on request.

## Supporting Information Available

Total and projected DOS of the Ta+Mg (Figure S1), TaN (Figure S2), TaO_0.5_N_0.5_+MgO_0.5_N_0.5_ (Figure S3), and MgN (Figure S4) models (PDF)
