# Supplementary material for: Digging into the Atomistic Details of the TaN/MgO Interface: An Ab Initio Study Supported by Transmission Electron Microscopy
Source: ACS Mater Au. 2025 Jan 28;5(2):421–9. doi: 10.1021/acsmaterialsau.4c00173 (PMC11907287; doi:10.1021/acsmaterialsau.4c00173)
Supplement: Supplementary file 1 — mg4c00173_si_001.pdf [file mg4c00173_si_001.pdf]

Supporting Information

Digging into the atomistic details of the TaN/MgO interface: an ab initio study supported by transmission electron microscopy

Victor Quintanar-Zamora[a,b], *Joseph P. Corbett*[c], *Rodrigo Ponce-Pérez*[b], *Armando Reyes Serrato*[b], *Carlos Antonio Corona-Garcia*[b], *Oscar Contreras-López*[b], *Jonathan Guerrero-Sanchez*[b], *and Jesús Antonio Díaz*[b]*.

[a] *Posgrado en Nanociencias, Centro de Investigación Científica y de Educación Superior de Ensenada, Baja California, Ensenada, Baja California, 22860, México.*

[b] *Centro de Nanociencias y Nanotecnología, Universidad Nacional Autónoma de México, Ensenada, Baja California, 22860, México.*

[c] *Department of Physics, College of Arts and Science, Miami University, 501 E High St, Oxford, Ohio, 45056, United States.*

**Corresponding author: olaf@ens.cnyn.unam.mx*

The total density of states (TDOS) and the projected density of states (PDOS) of the Ta+Mg, TaN, $TaO_{0.5}N_{0.5}$+$MgO_{0.5}N_{0.5}$, and MgN interface models are shown below. The $TaO_{0.5}N_{0.5}$+$MgO_{0.5}N_{0.5}$ is the only model in which the p_x and p_y orbitals of Ta, N, Mg, and O atoms, as well as the d_{xz} and d_{yz} orbitals of Ta atoms, are not degenerated.

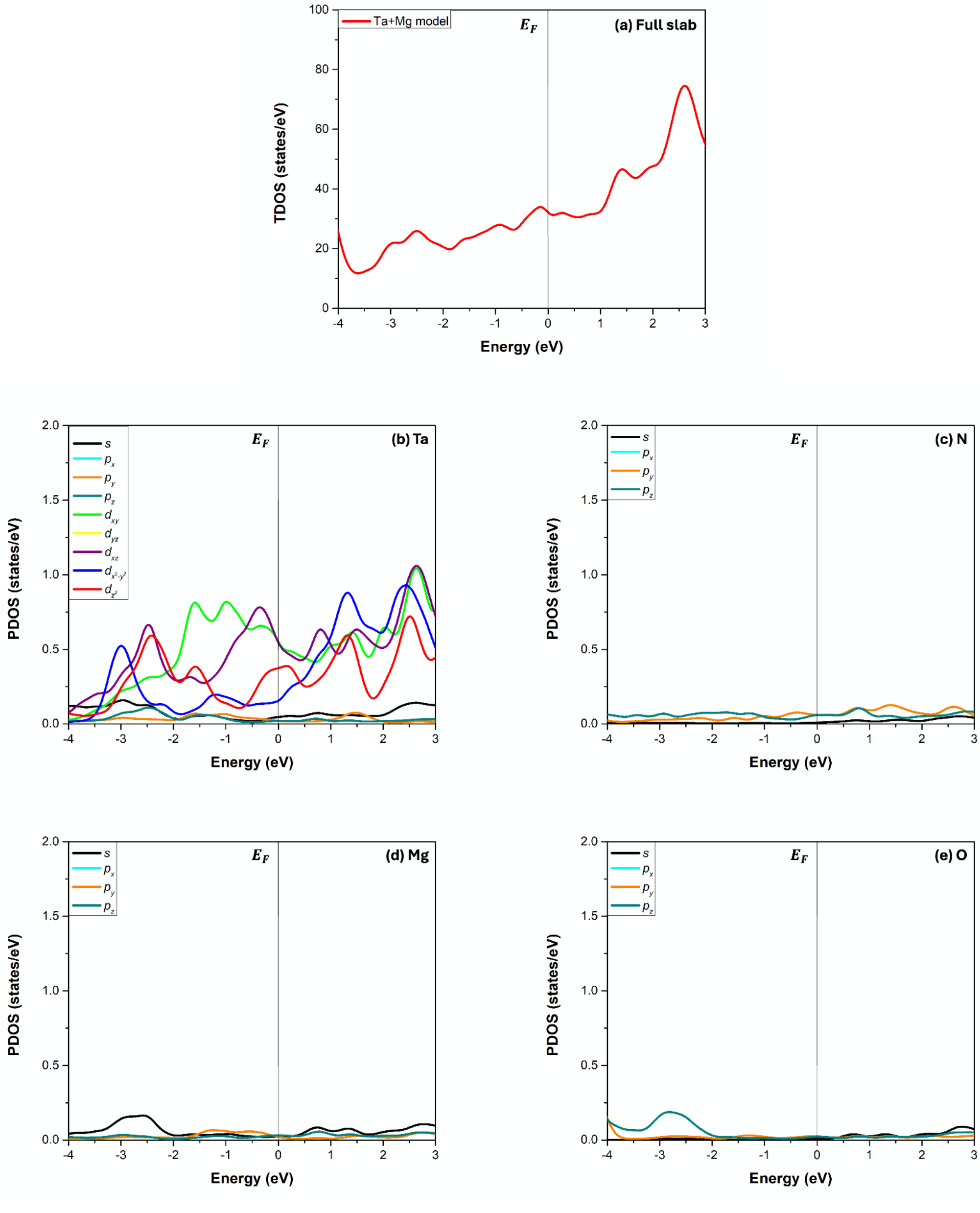

Figure S1. Density of states of the Ta+Mg model (a) total DOS of the full slab and projected DOS of the (b) Ta, (c) N, (d) Mg, and (e) O atoms at the interface layers.

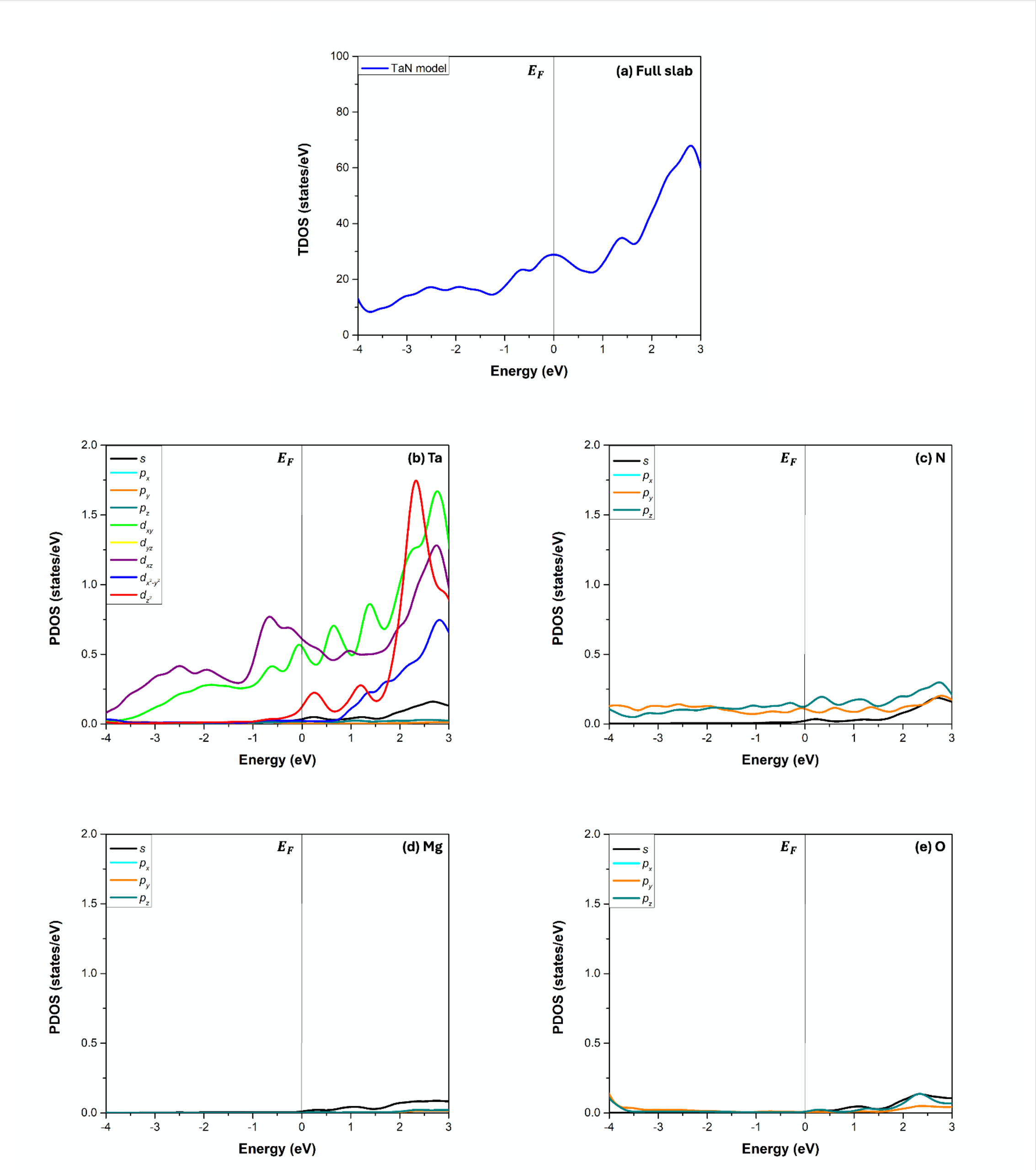

Figure S2. Density of states of the TaN model (a) total DOS of the full slab and projected DOS of the (b) Ta, (c) N, (d) Mg, and (e) O atoms at the interface layers.

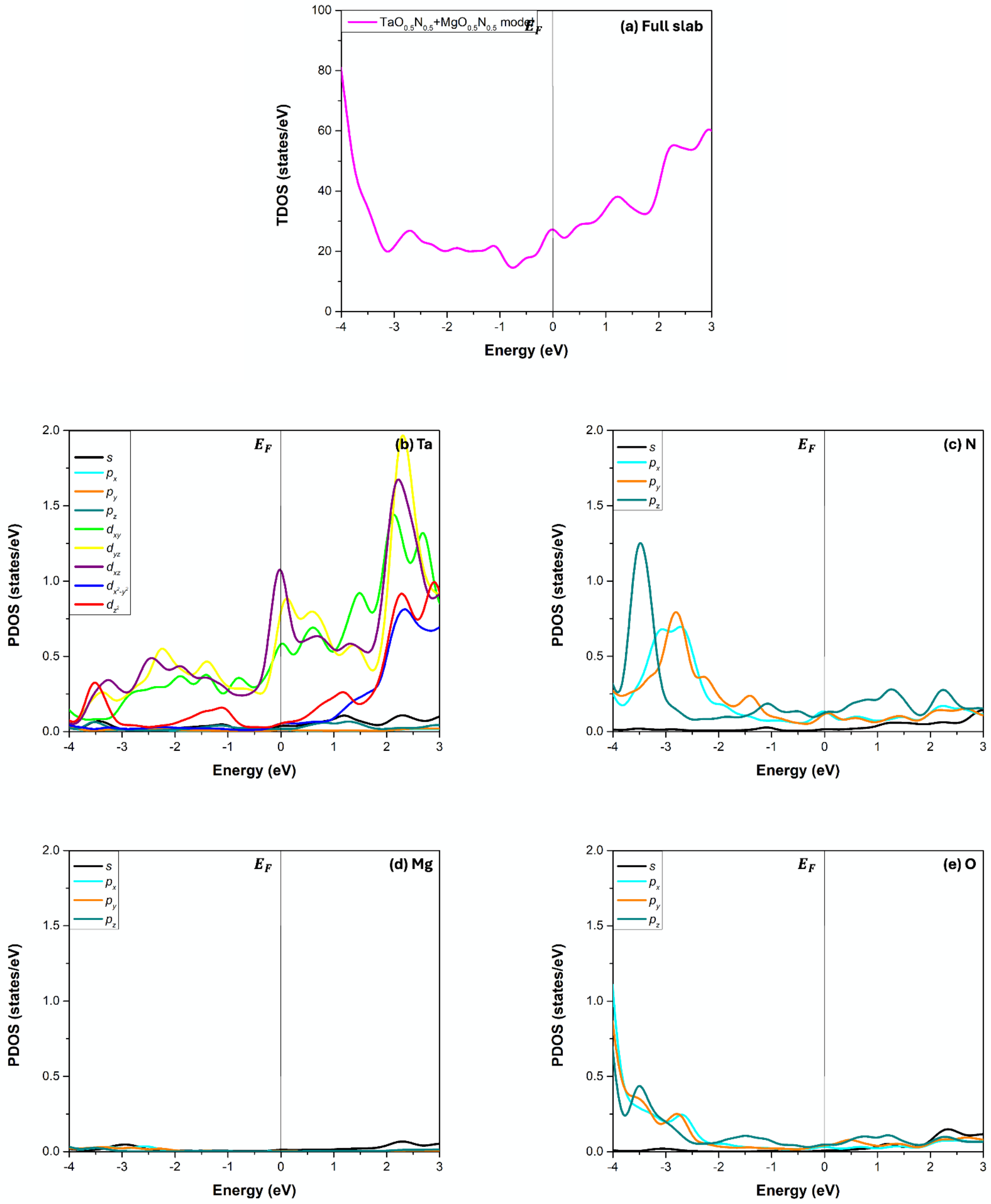

Figure S3. Density of states of the $TaO_{0.5}N_{0.5}$+$MgO_{0.5}N_{0.5}$ model (a) total DOS of the full slab and projected DOS of the (b) Ta, (c) N, (d) Mg, and (e) O atoms at the interface layers.

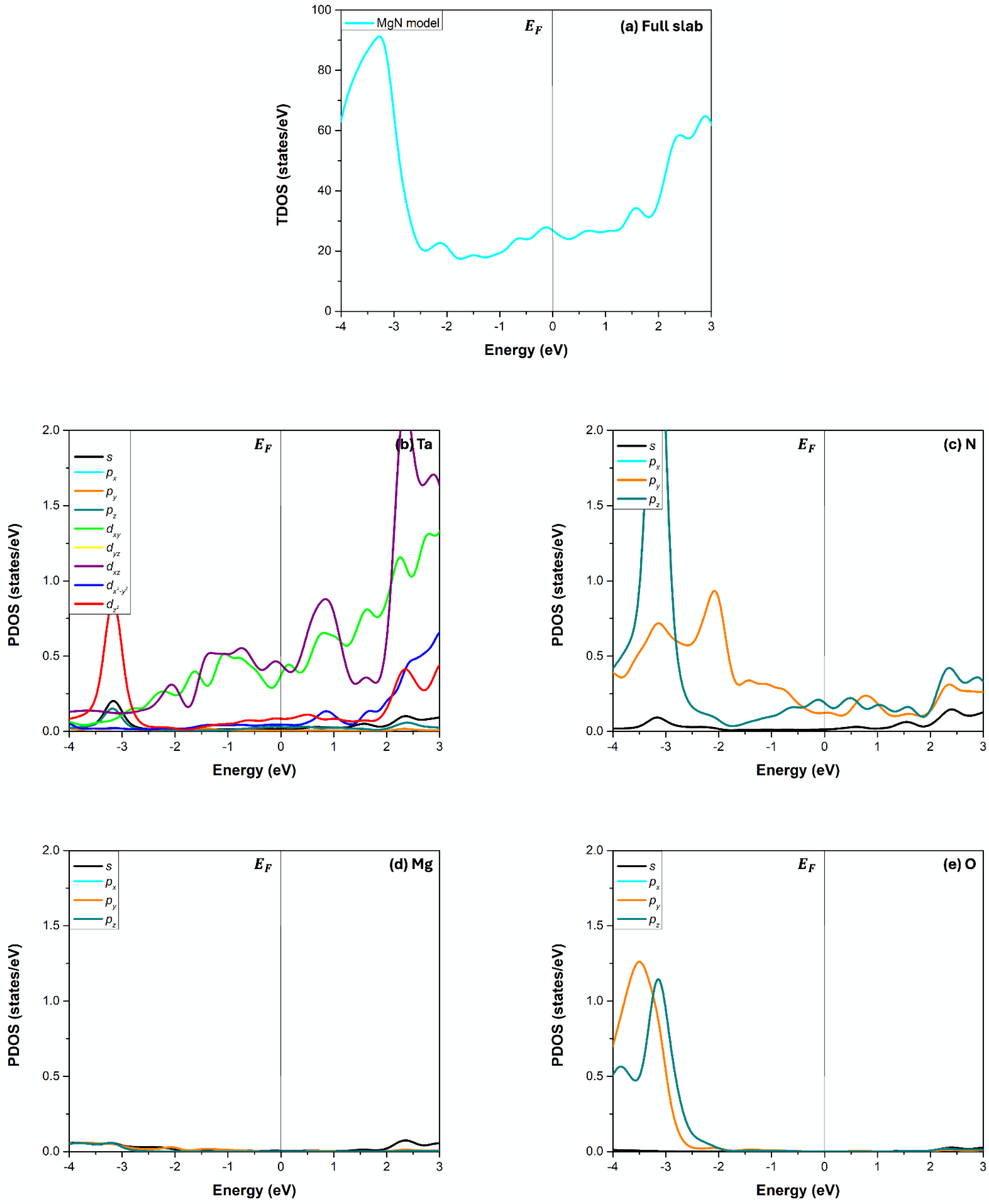

Figure S4. Density of states of the MgN model (a) total DOS of the full slab and projected DOS of the (b) Ta, (c) N, (d) Mg, and (e) O atoms at the interface layers.
